# Editorial: Aging and neurodegeneration in the brain

**DOI:** 10.3389/fragi.2025.1581161

**Published:** 2025-04-02

**Authors:** F. Kerr, K. J. Kinghorn, T. Niccoli, N. Woodling

**Affiliations:** ^1^ Centre for Biomedicine and Global Health, School of Applied Sciences, Edinburgh Napier University, Edinburgh, United Kingdom; ^2^ Department of Genetics, Evolution and Environment, Institute of Healthy Ageing, University College London, London, United Kingdom; ^3^ School of Molecular Biosciences, University of Glasgow, Glasgow, United Kingdom

**Keywords:** aging, brain, neurodegeneration, genomics, proteomics

In the ever-evolving landscape of neurodegeneration and aging research, uncovering the molecular mechanisms underlying these processes is essential for advancing strategies to promote healthy aging. The studies included in this Research Topic together offer complementary insights into these mechanisms, as well as uncovering molecular signatures that accurately predict biological age and closely associate with the development of aging-related diseases.

Mechmet et al. and Sohn et al. utilize functional genomic approaches to investigate the causal roles of specific genes in olfactory decline and neuronal degeneration. Meanwhile, Lobon et al. and Coenen et al. employ large-scale genomic and proteomic analyses to identify novel molecular landscapes associated with aging and specific neurodegenerative diseases, such as Parkinson’s disease (PD). Together, these studies enhance our understanding of the molecular mechanisms underlying age-related pathologies and neurodegeneration, laying the groundwork for future studies to determine their therapeutic potential for both prevention and treatment of neurodegenerative conditions.

Mechmet et al. explore age-related olfactory decline in microphthalmia-associated transcription factor (*Mitf*) mutant mice. MITF is a basic helix-loop-helix leucine zipper transcription factor that regulates immune function and neuronal homeostasis through transcriptional control of genes involved in lysosomal biogenesis and autophagy - central processes in microglia - and macrophage-driven inflammatory responses. While olfactory decline is a well-known marker of aging and an early sign of neurodegenerative disorders, this study examines whether *Mitf* deficiency leads to neurodegeneration and olfactory dysfunction with age. Interestingly, aging *Mitf* mutant mice exhibit olfactory decline compared to wild-type controls without evidence of neurodegeneration or major morphological or inflammatory changes in the olfactory bulb. Moreover, the researchers identified compensatory mechanisms, such as the upregulation of potassium channel subunits, as potential buffers against hyperexcitability, a precursor of neurodegeneration. These findings raise important questions about MITF’s role in neuronal stability across sensory systems during aging. The absence of degenerative phenotypes in aging mice, despite continued olfactory decline, underscores the complexity of sensory aging mechanisms and highlights the interplay between vulnerability and resilience in age-related sensory deficits.

Sohn et al. investigate mechanisms of age-associated neurodegenerative diseases through their study on the effects of tau mutations on lamin nucleoskeletal destabilisation, nuclear tension, and cellular mechanosensing. The Linker of Nucleoskeleton and Cytoskeleton (LINC) complex connects nuclear lamin intermediate filaments to cytoskeletal actin filaments, via Sad1, UNC84 (SUN)-domain proteins, and to microtubular motors via nesprins. Previous studies demonstrated that cells harboring pathogenic tau phospho-mutations exhibit destabilization and invagination of the lamin nucleoskeleton, and *Drosophila* models expressing mutations affecting actin polymerisation and LINC exhibit alterations in the nuclear architecture and tau-induced neurodegeneration. Sohn et al. explored the effects of these disruptions on the mechanical sensing properties of neuronal nuclei by measuring nesprin tension between the nucleus and cytoskeleton. Inducible expression of R406W Frontotemporal Dementia-associated tau in BE (2)C human neuroblastoma cells caused increases in nuclear membrane blebbing and nuclear mislocalization of the LINC proteins SUN1, SUN2 and Nesprin. Induction of pathogenic tau reduced nuclear tension, as observed by increased Förster resonance energy transfer (FRET) in cells expressing the FRET sensor TSmod inserted into a mini-Nesprin-2G gene, suggesting that these changes may regulate early nuclear responses to mechanical signals. Further research is required to establish the mechanisms regulating these effects, their relevance to conditions influenced by cerebrovascular-generated mechanical stress within complex brain architecture, and whether they reflect pathogenic or compensatory protective responses to DNA damage.

Lobon et al. explore the emerging hypothesis that somatic mutations contribute to sporadic cases of PD. By employing whole exome sequencing and developing a Combined Or Single sample MOSaicism (COSMOS) detection approach to computationally identify somatic mutations from bulk sequencing data, their study identified somatic single nucleotide variants (sSNVs) across four brain regions and blood samples from ten PD patients. sSNVs were enriched in genes related to synaptic and neuronal functions, such as *GRIP1* and *KCNK2*, ubiquitination, including *UBE2U,* and glucose transport, *DENND4A*, all of which have been linked to several neurodegenerative diseases, including PD and Alzheimer’s disease. These findings suggest that many sSNVs may have a developmental origin, contributing to brain mosaicism and potentially influencing PD pathology, and underscore the relevance of somatic mutations in the molecular landscape of sporadic PD. While the correlation between deleterious sSNVs and clinical manifestations remains an area for future exploration, these findings provide a robust framework for further investigations into the molecular underpinnings of PD.

Coenen et al. compile a plasma proteome of aging using bioinformatic approaches to integrate four large-scale human plasma SOMAscan proteomic datasets from individuals aged 16–95 years. They identified changes in levels of 273 proteins associated with aging across these cohorts, with 56 forming an interconnected protein-protein interaction subnetwork enriched in diverse functions, including growth factor binding, metabolic disease and TNF receptor activation. Creating proteomic clocks, they found that 15 of these aging-associated proteins were sufficient to accurately predict an individual’s biological age. Clustering aging proteins that follow a similar expression trajectory revealed that enrichment of these proteins aligned with aging-dependent alterations in specific biological functions including extracellular matrix, insulin-like growth factor and coagulation signalling cascades, as well as regulation of neurodevelopment and GABA receptor binding–processes relevant to nervous system function. Compared to the rest of the plasma proteome, aging-associated proteins were more frequently linked to diseases, such as heart failure, pneumonia, kidney disease, and dementia. GDF15 and TNFRSF1A/1B were associated with the highest number of diseases, while the top 10 proteins shared links to 32 conditions, suggesting that aging proteins may serve as broad biomarkers of multiple diseases. Overall, this study identified a plasma proteome signature that associates with aging across independent cohorts, correlates with disease presence, and pinpoints a subset of proteins capable of accurately predicting biological age.

Taken together, these studies exemplify the broad range of research approaches required to understand how aging predisposes the nervous system to some of the most costly and devastating diseases in modern medicine. Spanning powerhouse genetic studies in invertebrate models, meticulous characterisation of mouse models, and bioinformatic studies of both focused and broad human cohorts, these findings open new avenues for future investigation, with the ultimate goal of developing treatments to preserve brain health and reduce disease burden during ageing.

